# Effect of an interactive voice response system on self-management in kidney transplant recipients: Protocol for a randomized controlled trial

**DOI:** 10.1097/MD.0000000000014291

**Published:** 2019-02-08

**Authors:** Raheleh Ganjali, Zhila Taherzadeh, Mahin Ghorban Sabbagh, Fatemeh Nazemiyan, Fereshteh Mamdouhi, Hamed Tabesh, Shapour Badiee Aval, Reza Golmakani, Sayyed Mostafa Mostafavi, Saeid Eslami

**Affiliations:** a Department of Medical Informatics, Faculty of Medicine; b Neurogenic Inflammation Research Center; c Targeted Drug Delivery Research Center; d Kidney Transplantation Complications Research Center; e Department of Nephrology, Faculty of Medicine; f Complementary Medicine Research Center, Faculty of Traditional Medicine; g Department of Emergency Medicine, Doctor Shariati Hospital; h Pharmaceutical Research Center, Mashhad University of Medical Sciences, Mashhad, Iran; i Department of Medical Informatics, University of Amsterdam, Amsterdam, The Netherlands.

**Keywords:** IVRS, kidney transplant recipients, medication adherence, patient transplant knowledge, quality of life

## Abstract

**Introduction:**

Adherence to a complex and ongoing set of therapeutic recommendations significantly determines short and long-term outcomes after kidney transplantation (KT). Interactive voice response system (IVRS) is a novel phone-based platform which is potentially useful to deliver health behavior interventions.

**Objective:**

The aims of the study is to describe the development of a theory-driven and educational IVRS investigate the effect of an IVRS on the self-management outcomes in KT recipients as compared with the control group.

**Methods:**

This study has been designed as a randomized, 2 parallel groups controlled trial. The KT recipients’ older than18 years of age with access to a cellphone will be included. A total of 120 patients will be randomly assigned to the control and intervention groups. The participants in the intervention group will receive completely automatic calls in 3 categories: educational, medication adherence, and reminders by the IVRS, whereas those in the control group will receive usual care. The follow up will be performed within 6 months. The primary outcome will be the medication adherence while patients’ transplant knowledge, health-related quality of life, and rehospitalization rates will be considered as secondary outcomes.

**Results:**

Thus far, recruitment of participants has not been completed and results will be published in 2019.

**Discussion:**

The IVRS is potentially useful to help KT recipients improve the self-management outcomes. The hypothesis is using an IVRS intervention makes a significant difference between basel assessment of adherence to immunosuppressive medications scale, 12-item short form survey, second version, kidney transplant understanding tool baseline scores, and those obtained at the end of study.

**Trial registration number:**

This trial is registered with the Iran Trial Registrar under registration number IRCT20180124038492N1 and registration date 30 January 2018. https://irct.ir/trial/29215

## Abstract 2

Supplemental Digital Content is available in the text

## 1 Introduction

Kidney transplantation (KT) is a surgical replacement procedure to remove a healthy, functioning kidney from a living or deceased donor and implant it into a patient with end-stage renal disease. The short and long-term outcomes after KT are largely determined by the patient's adherence to a complex and ongoing set of medical recommendations, aimed at minimizing the risk of graft loss and comorbid complications.^[1,2]^ Self-management is intended to help patients to maintain wellness in their foreground perspective. Self-management programs must include content that addresses the following 3 tasks: medical or behavioral management, role management, and emotional management.^[3]^ For KT recipients, self-management tasks include adhering to an immunosuppressive regimen, monitoring for signs of major complications (ie, rejection, infection, and malignancies), keeping regular appointments with the nephrologists, and diet management.^[1,3,4]^

Previous studies have shown that non-adherence to immunosuppressant is associated with 60% increased risk of graft rejection among KT recipients.^[5]^ Moreover, a recent longitudinal study has proved that the patients with appointment non-adherence had 1.5 times higher risk of acute rejection and 65% higher risk of graft loss.^[6]^ Several observational studies have published high non-adherence rates to lifestyle recommendations.^[7,8]^ Kobus et al have shown that 85.3% of patients did not change their diet after KT and 64.2% were not aware of dietary recommendations.^[8]^ Adherence to recommended lifestyle is critical to maintain the positive prognosis as well as health-related quality of life (HRQoL). Employing self-management programs might be useful to help patients adapt a proper lifestyle.^[9]^

In developing countries, widespread use of mobile devices and almost universal coverage of signal network has given mobile-based interventions (m-Health) a higher opportunity to be more affordable, effective, and accessible among patients.^[10–12]^ It has been previously proved that m-Health interventions have become successful to obtain objectives in low-income countries in an economically viable and sustainable way.^[13,14]^ The m-Health interventions can be used to provide educational and supportive contents, reminders, and immediate response to patients’ questions aiming to overcome weak access to information and limited human resources.^[15–17]^

The integration of telephony with computers has created an advancement in health information technology called interactive voice response system (IVRS), which, in particular, has emerged in the form of many applications dealing with chronic disease management.^[16,18,19]^ Using IVRS, patients interact with a set of organized voice-recorded message components and are able to respond to queries using their touch-tone phones. The IVRS offers a way to help individuals address their health literacy issues. Since the per-contact cost of IVRS is low, frequent health monitoring messages can be sent to ensure continuous contact with patients.^[20]^ In addition, since patients carry their mobile phone with them wherever they go, the occurrence of missing or unsuccessful calls are very unlikely.^[18]^

Many IVRS applications in healthcare have recently been explored. For instance, a recent review on the effectiveness of IVRSs for managing chronic conditions concluded that these interventions are useful to change patients’ behaviors and, consequently, improve health-related outcomes.^[21]^ Furthermore, other studies have shown that patient education is the best way to effectively improve medication adherence. Depending on the type of non-adherence and the patient's profile, the use of a combination of educational interventions (eg, patient education, self-care, and patient motivation) has the greatest potential to improve medication adherence.^[22–25]^

Health education programs are aimed to improve outcomes, increase the benefits for the community, and, consequently, enhance public health.^[26]^ More specifically, the program logic model (PLM) helps to identify the measurable outcomes in the causal chain.^[27]^ PLM offers a framework to guide the content of e-health education programs, enabling the healthcare providers to more effectively define and measure objectives, empower patients, and improve patient care.^[28]^ The PLM will be used during the development and implementation phases of this study. According to the PLM, patient education results in proximal outcomes (eg, knowledge and compliance) and intermediate outcomes (eg, self-efficacy and QoL). As a long-term effect, patient education programs might decrease or eliminate the loss of productivity, increase community capacity, and increase the efficiency of health care resources. Since post-transplant management is a complex process and KT recipients have a lot to learn, inadequate patient knowledge can easily result in dire consequences (eg, decreased adherence to recommendations and organ rejection).^[29,30]^ We hypothesized that automatic IVRSs designed based on educational and theory-driven intervention which is acceptable to the individual patient would improve self-management in KT recipients who received IVRS compared to patients who only received usual care. Thus, we designed a randomized, 2 parallel groups controlled trial to evaluate the effect of an IVRS intervention on self-management outcomes (such as immunosuppressant medication adherence, transplant knowledge, and quality of life) among KT recipients.

## 2 Theoretical framework

Behavior and behavior change are described as the critical components of a self-management process for chronic diseases.^[33]^ According to Barlow and colleagues, self-management is defined as the ability of the patient to manage the symptoms of complications, treatment approach, physical and psychosocial issues, and lifestyle patterns.^[34]^

The intervention in this study was based on the self-regulation theory,^[35]^ which describes how patients understand their illness and how they establish coping strategies to manage their condition.^[36]^ In this theory, while a patient is considered as an “active problem-solver,” the role of both cognitive and emotional processes determines the illness perceptions and associated coping strategies (ie, self-management behaviors).^[36]^ Moreover, the self-regulation theory suggests that relevant factors should be displayed by the content of interventions including accurate information, creating behavioral skills and supplying effective support.^[37]^ This theory focuses on the patient's personal model to determine his/her behavioral responses to illness. Five core elements are identified for transplant representations: identity, cause, timeline, consequences, and treatment effectiveness.^[38]^ Some environmental modifications can be used to enhance the self-regulation process, such as the reminder systems. These systems promote the patient's self-efficacy by eliminating the barrier of forgetfulness.^[39]^ Cost-effectiveness of such systems helps researchers to manage patient adherence.^[40,41]^

## 3 Methods

### 3.1 Design and development of IVRS

We used the following phases to design and develop our IVRS:

#### 3.1.1 Preliminary study to investigate patient requirements

In a preliminary study, non-adherent immunosuppressive patients were determined by basel assessment of adherence to immunosuppressive medications scale (BAASIS).^[31]^ Also, in this group of patients, barriers to medication non-adherence were determined by the immunosuppressive therapy barriers scale.^[32]^ Our investigation in patient requirement identified the barriers to adherence as follows: use of multiple pills of immunosuppressive medications at 1 time, lack of understanding about the usefulness of immunosuppressive medications, confusion about medication taking, and difficulty in remembering the medication taking.

#### 3.1.2 Educational content

An expert group was created, consisting of a group of nephrology specialists, patient education specialists, nurses, and medical informatics specialists. Three focus group sessions with 10 individuals were formed to determine provider expectations of these patients over a period of 8 hours. In this focus group, the booklet related to transplant patients was reviewed and educational content was corrected and confirmed according to the patient requirements and provider expectations. Educational content consisted of the following 7 sections:

(1) Immune system and its role in rejection.
(2) Immunosuppressive medications and complications.
(3) Infections and methods for prevention.
(4) Nutrition.
(5) Long term care.
(6) Return to work and life.
(7) Sexual activities and pregnancy.

Each section consisted of between 2 and 8 subsections.

An additional file shows more details about the export group, (see Supplemental file 1).

#### 3.1.3 Patient profile

A Delphi round by the expert group determines the data elements needed for the patient profile. The final profile of the patient consists of 5 main categories, including:

(1) Demographic information (unique number, age, gender, educational level, mobile phone number, city, type of residence, and patient address)
(2) Medical information (date of transplant, donor)
(3) Type of immunosuppressive medication and usage intervals,
(4) Patient's passcode
(5) Date of referral and patient's follow up.

We need the information in the profile to make individualized IVRS's calls and reminders.

#### 3.1.4 IVRS development

The IVRS consisted of several technologies working together in order to schedule, receive, enter, and record automated phone calls. We used the Linux Cent operating system, VoIP network, PHP as the Programming language to write IVR call flow/script and MySQL server as a database that connected to the Asterisk server. IVRS is implemented by installing the Asterisk server on the host machine and creating virtual machines. One e1 line is connected to the VoIP network to transfers out the phone calls. For each patient, all patient profile information and setting calls must be entered. Also, every interaction of the system with the patient will be recorded in the patient profile as patient's reports. Patient reports also contain the date, time and content of short messages service (SMSs) and phone calls.

**Figure 1:**
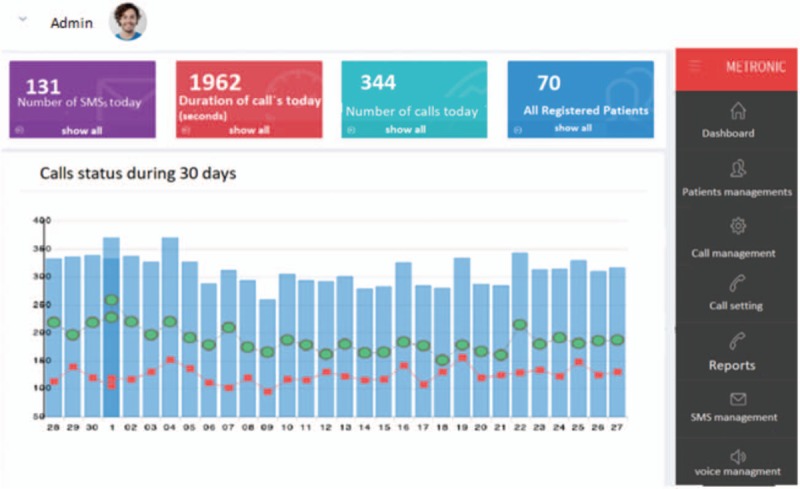
A screenshot of the web application for managing the IVRS. IVRS = interactive voice response system.

Figure 1 shows a screenshot of the web application for managing our proposed IVRS. The boxes at the top of the figure show some important statistics such as the number of calls and SMSs. The diagram reveals calls’ status in the previous 30 days, where blue bars show the number of all calls in that day, green nodes indicate the number of completed calls, and red nodes represent the number of missed calls. The main menu at the left of the figure navigates the administrator of the IVRS to other pages of the application. It is worth noting that the only user of this application is the administrator of the IVRS. An additional file shows more details about the application, (see Supplemental file 2).

#### 3.1.5 Pilot implementation of IVRS

Information on 10 KT recipients (who were not considered for final evaluation) was entered into IVRS with preset reminders and educational calls. After 2 weeks, the same 10 participants were called and asked some questions about their experience with the working with the system, particularly focusing on their satisfaction, ease of use and possible problems with the system. Ninety percent of KT recipients expressed that they were satisfied and interested in the sent reminders and educational calls. An additional file shows more details about the questions, (see Supplemental file 3).

### 3.2 Evaluation IVRS

#### 3.2.1 Study design

**Figure 2:**
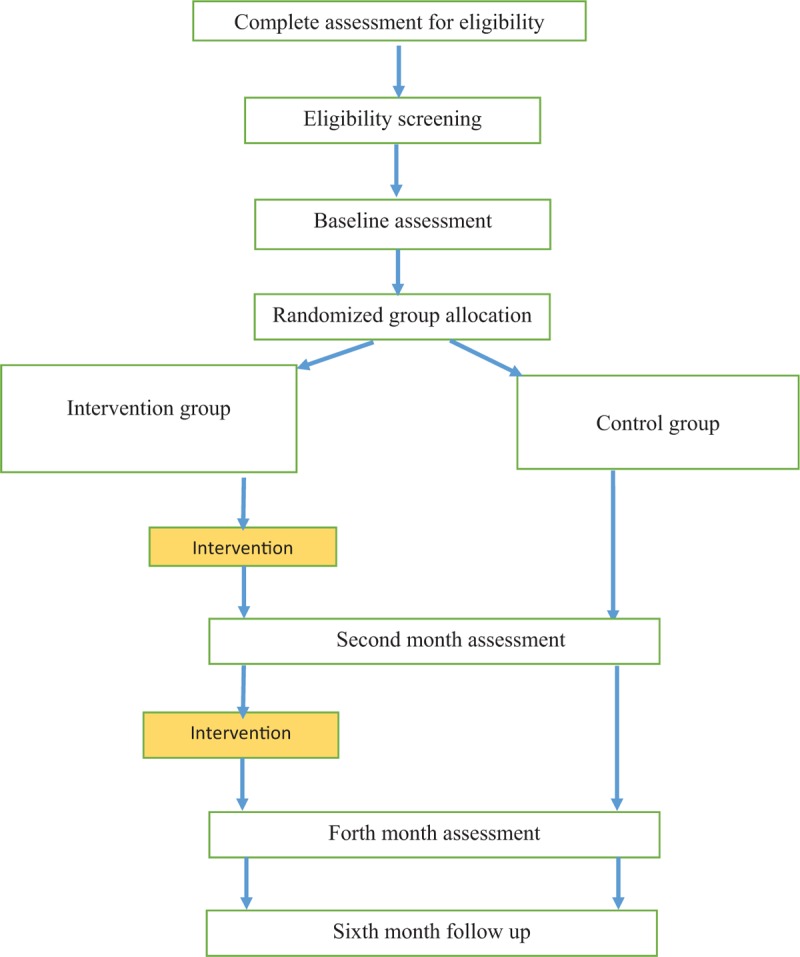
Consort diagram.

A randomized, 2 parallel groups, controlled trial in accordance with the Consolidated Standards of Reporting Trials guidelines^[33]^ was designed (Fig. 2). The protocol is reported in accordance with the Standard Protocol Items: Recommendations for Interventional Trials 2013 Statement.^[34]^

#### 3.2.2 Study setting

The study will be conducted in Montaserieh Transplant Center in Mashhad, Khorasan Razavi province, northeastern Iran. Montaserieh is the only specialized governmental referral educational hospital providing organ transplantation in northeastern Iran. The center is affiliated with Mashhad University of Medical Sciences (MUMS).

### 3.3 Participants

KT recipients who will be referred to the center in July 2018 for follow up visits and caretaking are screened for eligibility. Eligible patients will be asked to provide consent with appropriate trail information and enough time to study and ask questions. Patients will be received 2 types of information related to the study:

(1) fully understandable oral information
(2) 2informed consent, in which the study design is described completely.

Informed consent has already been evaluated by the Ethics Committee of MUMS (IR.MUMS.fm.REC.1396.160). We ensure participants can withdraw from the study at any time without any commitment and no effect on their subsequent care. Eligible participants will be included after completing informed consent.

The inclusion criteria are as follows:

(1) KT recipients who have received a KT between 20 March 2012 and 20 March 2017,
(2) having 18 years of age or more,
(3) having acceptable graft function (do not need any types of dialysis),
(4) having access to a mobile phone,
(5) being able to read the informed consent information, and
(6) willingness to participate.

The patients who are refused to participate in the study will be excluded. KT patients will be randomly assigned to the control (usual care) or intervention (usual care plus IVRS) groups. Usual care includes an educational booklet for KT and patient education provided by the nephrologists.

### 3.4 Interventions

Three types of completely automatic calls will be received by KT patients through the IVRS.

#### 3.4.1 Reminder call

The purpose of a reminder call is to remind the patient about the time for taking immunosuppressive medications and periodic appointment. Medication reminder calls will be done once a day based on the type of immunosuppressive medications which are recorded in the patient profile. The IVRS will contact the patient's mobile number and remind them about taking their medication. In the absence of an answer, the system will call again 30 seconds later, and if she\he does not respond, it will send a text message containing the medication reminder.

The periodic appointment reminder calls will be done every 2 months based on the date of referral which is recorded in the patient's profile a day before their appointment. Moreover, on the day of appointment, patients will receive a reminder in the form of an SMS.

#### 3.4.2 Educational calls

Educational calls, which contain information about self-management education in KT patients, are made every day based on educational content to improve patient transplant knowledge. These messages are recorded with a clear voice. This part of IVRS is patient-centered, whereby calls are made at specific times determined by the patient. Entering contacts menu will be created so that KT patients can dial 31,806 and after record passcode, they can access to classified educational information.

#### 3.4.3 Medication adherence call

Medications adherence calls measure immunosuppressive adherence on a daily basis. These calls are designed to encourage the patient to answer 2 questions at the end of the day. The first question is concerned with taking the immunosuppressive medication and the second one is about delayed timing. The participant is asked to answer the questions using the phone keypad. Answers include 0 = never, 1 = once, 2 = twice, 3 = more than twice. Patients selecting an answer other than “never’” were considered as non-adherent in that day. Every 10 days, the system sends a feedback based on patient's answers. Feedbacks will be provided in the form of an SMS. The content of this message will reveal the status of patient adherence (very poor, poor, moderate, good, and excellent) and will contain a motivating message.

Participants can withdraw from the study at any time and for any reason. The researchers can remove a participant from the study if they miss more than 2 follow-up visits or if they need some type of dialysis based on a nephrologist's expert opinion.

### 3.5 Outcomes measure

Study outcomes will be evaluated in accordance with Table 1. Primary and secondary outcomes are measured at baseline, and at 2-months, 4-months, and 6-months after baseline. For ethical reasons, if the intervention is found to be effective, all groups will have access to IVRS at the end of the intervention. Primary outcome will be immunosuppressive medication adherence that is evaluated based on a self-report in both groups during the follow-up period. The secondary outcomes include transplant knowledge, HRQoL, and rehospitalization rate.

**Table 1:**
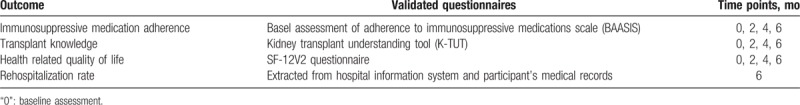
Timing of outcome measurement and validated instrument.

### 3.6 Sample size

According to the study by Garcia et al,^[35]^ an study effect size based on 90% power and data with a 2-tailed *α* = 0.05, in medication adherence between the control and intervention groups, was estimated to be 40 patients in each group. Considering the 50% prop up rate during the study, our goal is to employ 60 patients in each group.

### 3.7 Participant timeline

Participants’ timeline will be done based on Table 1. In the baseline assessment, we asked patients about the suitable timing to receive educational calls.

### 3.8 Randomization

After reviewing the outpatient medical records of patients referred to the clinic, we first obtain their informed consent. Then participants will be stratified into different strata based on their respective date of transplantation. The first stratum will be included participants who have received a transplant between March 21, 2015 and March 20, 2017, and the second stratum will include recipients who have received a transplant between March 20, 2012 and March 20, 2015. In fact, the goal is to eliminate the effect of post-transplantation time in evaluating self-management patients. Then participants will be randomly assigned to the control or intervention group in each stratum. For generating the allocation sequence, we will be used computer-generated random numbers provided by www.randomization.com. Randomization sequence will be created by an independent researcher, who is not involved in the collection or intervention procedure. The allocation ratio of intervention: control is 1:1.

For each patient, a folded paper, containing the assignment group together with a unique identification number in the study, will be stored in a sealed envelope, such that the information will not be readable without opening the envelope. Sealed envelopes are arranged based on randomized sequences. The assistant researcher will collect the baseline questionnaire and assigns the patient to the relevant stratum. Subsequently, the assistant researcher contacts the researcher (responsible for registering the patient in the IVRS) and the researcher will assign the patient to 1 of the 2 intervention or control groups. If the participant is assigned to the intervention group, the researcher registers the participant in the IVRS based on baseline information.

### 3.9 Blinding

Because of the nature of the study, the allocation of participant to the control or intervention group cannot be completely blind. However, physicians and outcome evaluators will be completely blinded to the control and intervention group. Hospital staff who provide clinical and educational care and treatment services to patients will also be blinded to the allocated group. Researchers other than those in the clinical trial team and the research assistant (who will register the participants and evaluate the baseline) will be blinded to the group allocation throughout the study.

### 3.10 Data collection tools

BAASIS^[31]^ is a self-report tool containing 4 items for measuring adherence to immunosuppressive medications over the past 4 weeks. This tool has important dimensions such as non-adherence to medication (missing a dose), continuous missing of several doses, non-adherence to medication taking time (deviation more than 2 hours from the exact time), and reducing the doses. The patients were asked to respond on a 6-point Likert scale including 0 = *never*, 1 = *once a month*, 2 = *every 2 weeks*, 3 = *every week*, 4 = *more than once a week*, and 5 = *every day*. Patients selecting an answer other than “never” were considered as non-adherent. This tool was translated into Persian using the forward–backward translation method. Then another specialist compared the new version with the old version, and the incompatibilities were removed. The revised version was ultimately approved by the translators, and the final draft was prepared.

Patients’ transplant knowledge treatment process will be measured using the kidney transplant understanding tool (K-TUT).^[29]^ This questionnaire was first translated to Persian by 2 researchers (forward) and then back-translated by 1 individual (backward). Questionnaire's content validity was checked through 1 round of a Delphi session with the participation of 4 nephrologists and 4 coordinator nurses. To assess content validity quantitatively, the content validity index (CVI) and content validity ratio (CVR) indices were used. CVI was computed as the number of experts giving a rating 3 or 4 to the relevancy of each item, divided by the total number of experts. In order to determine CVR, the experts were asked to examine each item based on a 3-part spectrum: it is “necessary,” “useful,” and “not necessary,” Then the responses were evaluated according to the formula (the number of specialists selecting the “necessary” option divided by the total number of the specialists). Items with CVI values less than 0.78 were deleted. The questionnaire reliability was checked through test-retest reliability analysis. The questionnaire was given to 25 recipients. After a week they were asked again to answer the questions, and Pearson Correlation Coefficient was calculated for patient's transplant knowledge scores. This coefficient was 0.72, indicating the questionnaire acceptable level of reliability. The final questionnaire contained 9 true/false and 13 multiple choice questions (more than 1 correct answer). One score was given to each correct answer. The maximum knowledge score was 57. Higher knowledge scores indicated more KT knowledge.

In order to assess the health-related quality of life, the 12-item short form survey, second version (SF-12V2) questionnaire was administered. This questionnaire was validated by Montazeri et al for a group of Iranian patients.^[36]^ This 12-item questionnaire evaluates a general understanding of your health, physical performance, physical health, emotional problems, physical pain, social function, vitality and vital energy, and mental health. The maximum quality of life score is 48. A score greater than 37 indicates a good quality of life, a score between 25 and 36 indicates intermediate quality of life, and a score less than 24 indicates poor quality of life.

Rehospitalization was defined as any unscheduled rehospitalization (our program does not hospitalize for routine follow-up evaluation). The total number of rehospitalization days over the duration of the study, as measured by the cumulative number of hospitalization days over this time period, were extracted from the hospital information system and participants’ medical records.

### 3.11 Data management

Any decisions about outputs and decisions from this trial should be ratified by the trial Management Committee (SE, MGS, FN, FM, ZT, SBA, RG, and RG) and each of the named investigators shall be eligible to have authorship. All data management should be overseen for quality by our management committee. Data entry and coding of the deidentified data will be conducted by trained staff and we will use range checks for data values.

### 3.12 Statistical analysis

For participants in the 2 groups, the intervention and control, medication adherence will be measured and documented based on the BASSIS self-report at baseline, 2, 4, and 6 months. For determining effect of each predictor variable and modifying the effect of confounding, we will use ordinary logistics regression and multinomial logistics regression models. To evaluate quantitative variables at different time points, if the variables have a Gaussian distribution, the repeated ANOVA measure, and otherwise the Friedman test, will be used. Cochrane test will be used to evaluate and compare medication adherence across different time points. *P*-values less than .05 will be considered as statistically significant. All statistical analyzes will be performed using the Statistical Package for the Social Sciences, version 23, IBM. The electronic data file is stored on secure data servers and its hard copy version will be stored in a locked file at MUMS.

### 3.13 Research ethics approval

This study was approved by the Ethics Committee of MUMS and Medical School (IR.MUMS.fm.REC.1396.160, 2017-08-13).

### 3.14 Confidentiality

Participants’ personal information will be kept separate from the main dataset and will not be made publicly available. It will be stored on a secure file server research drive at MUMS to ensure confidentiality protection before, during and after the study.

## 4 Results

We have complete preliminary qualitative and expert opinion studies that provide educational content, profile information, and intervention design. Recruitment of participants has not been completed and results will be published in 2019. The IVRS is potentially useful to help KT recipients improve self-management outcomes. Using IVRS intervention will make significant differences between BAASIS, SF-12V2, K-TUT scores, when comparing these at baseline and at the end of study.

## 5 Discussion

IVRS has been used in different parts of health care including after hospital discharge to encourage test adherence, medication adherence in patients with asthma and human immunodeficiency virus, heart disease and self-management in back pain and post-stroke.^[37–43]^ IVRSs are used in medication adherence in different conditions with different functions.^[5,41,44–47]^ Previous studies showed that providing information about the health consequences appeared effective in promoting medication adherence. Such information usually includes the advantages of taking medications or the disadvantages of not taking them.^[42,48]^ In this study, we tried to design an intervention based on barriers for immunosuppressant medication adherence. We will evaluate the effect of educational, reminder, and feedback interventions not only in immediate outcomes such as knowledge and medication adherence, but also on intermediate outcomes such as quality of life and follow-up visits.

An important aspect of our study is that it is based on a behavior change theory. Theory-based interventions can make effective changes in behavior.^[6–8]^ The incentives might be used to reduce intentional and unintentional barriers. We hypothesized that feedback with an incentive message might be more effective than other forms of incentives. Thus the RCT will be conducted in KT recipients to evaluate the effects of the IVRS on improvement of patient self-management. In particular, m-health interventions provide tangible incentives in the form of feedback, which have the potential to warn patients about their health conditions. The interactive elements in the IVRS can potentially help providers to realize the patient self-management or measurement of patient-reported outcomes and examine the process of behavioral changes.

One of the limitations of present is that behavioral change observed during any controlled study may not be directly translated into clinical practice. Change in adherence behavior reached over the period of the study may not be maintained after the program ends. Moreover, an automated program may not effectively satisfy the requirements and needs of all groups of patients.

To be able to effectively use the results of the present study in clinical settings, we may need a preliminary evaluation to better target the recipients of such an intervention. As such, for future studies it may be desirable to examine whether new transplant recipients or non-adherent patients respond differently to this type of intervention than those with longer transplant histories or those who have a better record of medication adherence in the past.

## Acknowledgments

We thank the National Institute for Medical Research Development for supporting this study as part of a PhD thesis for Raheleh Ganjali.

## Author contributions

SE is the principal investigator of the study and procured funding. RaG registered the trial. SE, RaG, ZT, HT, SBA, and ReG were responsible for the concept/idea/research design. MGS, FN and FM adapted are responsible for treatment integrity. SE and RaG were responsible for the randomization scheme and HT was responsible for data analysis plan. RaG and ReG are responsible for participant enrolment and data collection in collaboration with MGS, FN, and FM. RaG, ZT, and ReG drafted the manuscript and all authors critically revised the manuscript for important intellectual content. All authors read and approved the manuscript.

**Conceptualization:** Saeid Eslami, Zhila Taherzadeh, Shapour Badiee Aval.

**Data curation:** Raheleh Ganjali, Reza Golmakani, Mahin Ghorban Sabbagh, Fatemeh Nazemiyan, Fereshteh Mamdouhi.

**Formal analysis**: Saeid Eslami, Raheleh Ganjali, Hamed Tabesh.

**Funding acquisition:** Saeid Eslami, Shapour Badiee Aval.

**Investigation:** Saeid Eslami, Raheleh Ganjali, Zhila Taherzadeh, Mahin Ghorban Sabbagh, Fatemeh Nazemiyan, Fereshteh Mamdouhi, Hamed Tabesh.

**Methodology:** Saeid Eslami, Raheleh Ganjali.

**Project administration:** Raheleh Ganjali, Reza Golmakani, Mahin Ghorban Sabbagh, Fatemeh Nazemiyan, Fereshteh Mamdouhi, Saeid Eslami.

**Resources**: Saeid Eslami, Raheleh Ganjali.

**Software:** Raheleh Ganjali, Saeid Eslami.

**Supervision:** Mahin Ghorban Sabbagh, Fatemeh Nazemiyan, Fereshteh Mamdouhi, Shapour Badiee Aval, Saeid Eslami.

**Validation:** Saeid Eslami, Raheleh Ganjali, Hamed Tabesh, Reza Golmakani.

**Writing – original draft:** Raheleh Ganjali, Zhila Taherzadeh, Reza Golmakani.

**Writing – review and editing:** Raheleh Ganjali, Zhila Taherzadeh, Mahin Ghorban Sabbagh, Fatemeh Nazemiyan, Fereshteh Mamdouhi, Hamed Tabesh, Shapour Badiee Aval, Reza Golmakani, Sayyed Mostafa Mostafavi, Saeid Eslami.

## Supplementary Material

Supplemental Digital Content

## References

[1] Gordon EJ Prohaska T Siminoff LA Can focusing on self-care reduce disparities in kidney transplantation outcomes? Am J Kidney Dis 2005;45:935–40. 15861361 10.1053/j.ajkd.2005.02.011 PMC1249519

[2] Jamieson NJ Hanson CS Josephson MA Motivations, challenges, and attitudes to self-management in kidney transplant recipients: a systematic review of qualitative studies. Am J Kidney Dis 2016;67:461–78. 26372087 10.1053/j.ajkd.2015.07.030

[3] Low JK Williams A Manias E Interventions to improve medication adherence in adult kidney transplant recipients: a systematic review. Nephrol Dial Transplant 2014;30:752–61. 24950938 10.1093/ndt/gfu204

[4] Pinsky B Takemoto SK Lentine KL Transplant outcomes and economic costs associated with patient noncompliance to immunosuppression. Am J Transplant 2009;9:2597–606. 19843035 10.1111/j.1600-6143.2009.02798.x

[5] Andersson C Comparison of WEB and interactive voice response (IVR) methods for delivering brief alcohol interventions to hazardous-drinking university students: a randomized controlled trial. Eur Addict Res 2015;21:240–52. 25967070 10.1159/000381017

[6] Taber DJ The Impact of Health Care Appointment Non-Adherence on Graft Outcomes in Kidney Transplantation. Am J Nephrol 2017;45:91–8. 27907919 10.1159/000453554 PMC5214896

[7] Moller AC Merchant G Conroy DE Applying and advancing behavior change theories and techniques in the context of a digital health revolution: proposals for more effectively realizing untapped potential. J Behav Med 2017;40:85–98. 28058516 10.1007/s10865-016-9818-7 PMC5532801

[8] Dallery J Kurti A Erb P A new frontier: integrating behavioral and digital technology to promote health behavior. Behav Anal 2015;38:19–49. 27347477 10.1007/s40614-014-0017-y PMC4883489

[9] Bain-Brickley D Butler LM Kennedy GE Interventions to improve adherence to antiretroviral therapy in children with HIV infection. Cochrane Database Syst Rev 2011;12:CD009513. 10.1002/14651858.CD009513 PMC6599820 22161452

[10] Balke W-T Handels H Kalet IJ Discussion of spatial-symbolic query engine in anatomy’. Methods Inf Med 2012;51:479–88. 10.3414/ME11-01-0047 22614739

[11] van Velthoven MH Car J Zhang Y mHealth series: New ideas for mHealth data collection implementation in low- and middle-income countries. J Glob Health 2013;3:020101 doi: 10.7189/jogh.03.020101. 24363911 10.7189/jogh.03.020101 PMC3868820

[12] Yousef AM Chatti MA Schroeder U The state of MOOCs from 2008 to 2014: A critical analysis and future visions. InInternational conference on computer supported education 2014 Apr 1 (pp. 305–327). Springer, Cham.

[13] Cho YM Lee S Islam SMS Theories applied to m-health interventions for behavior change in low- and middle-income countries: a systematic review. Telemed J E Health 2018;24:727–41. 29437546 10.1089/tmj.2017.0249 PMC6205046

[14] Cormick G Kim NA Rodgers A Interest of pregnant women in the use of SMS (short message service) text messages for the improvement of perinatal and postnatal care. Reprod Health 2012;9:9. 22866753 10.1186/1742-4755-9-9 PMC3453517

[15] Dew MA Roth LH Thompson ME Medical compliance and its predictors in the first year after heart transplantation. J Heart Lung Transplant 1996;15:631–45. 8794030

[16] Kraft MR Androwich I Interactive voice response technology: a tool for improving healthcare. InNI 2012: Proceedings of the 11th International Congress on Nursing Informatics 2012 (Vol. 2012). American Medical Informatics Association. PMC3799157 24199090

[17] Sondaal SF Browne JL Amoakoh-Coleman M Assessing the effect of mHealth interventions in improving maternal and neonatal care in low-and middle-income countries: a systematic review. PloS One 2016;11:e0154664. 27144393 10.1371/journal.pone.0154664 PMC4856298

[18] Piette JD Striplin D Marinec N A randomized trial of mobile health support for heart failure patients and their informal caregivers: impacts on caregiver-reported outcomes. Med Care 2015;53:692. 26125415 10.1097/MLR.0000000000000378 PMC4503477

[19] Tsoli S Sutton S Kassavou A Interactive voice response interventions targeting behaviour change: a systematic literature review with meta-analysis and meta-regression. BMJ Open 2018;8:e018974. 10.1136/bmjopen-2017-018974 PMC5855236 29478016

[20] Butler JA Roderick P Mullee M Frequency and impact of nonadherence to immunosuppressants after renal transplantation: a systematic review. Transplantation 2004;77:769–76. 15021846 10.1097/01.tp.0000110408.83054.88

[21] Posadzki P Mastellos N Ryan R Automated telephone communication systems for preventive healthcare and management of long-term conditions. Cochrane Library 2016. 10.1002/14651858.CD009921.pub2 PMC6463821 27960229

[22] Conn VS Hafdahl AR Cooper PS Interventions to improve medication adherence among older adults: meta-analysis of adherence outcomes among randomized controlled trials. Gerontologist 2009;49:447–62. 19460887 10.1093/geront/gnp037

[23] Graves MM Roberts MC Rapoff M The efficacy of adherence interventions for chronically ill children: a meta-analytic review. J Pediatr Psychol 2009;35:368–82. 19710248 10.1093/jpepsy/jsp072

[24] Ross SM Role for automated communication strategies in medication adherence management. Am Health Drug Benefits 2008;1:20. PMC4106544 25126263

[25] Williams A Manias E Walker R Interventions to improve medication adherence in people with multiple chronic conditions: a systematic review. J Adv Nurs 2008;63:132–43. 18537843 10.1111/j.1365-2648.2008.04656.x

[26] Osborne RH Spinks JM Wicks IP Patient education and self-management programs in arthritis. Med J Aust 2004;180:S23. 14984359 10.5694/j.1326-5377.2004.tb05909.x

[27] Osborne RH Elsworth GR Whitfield K The health education impact questionnaire (heiQ): an outcomes and evaluation measure for patient education and self-management interventions for people with chronic conditions. Patient Educ Couns 2007;66:192–201. 17320338 10.1016/j.pec.2006.12.002

[28] Ceniceros X Testing the Effects of the E-Health Amputee Patient Empowerment Program (EAPEP) (Doctoral dissertation).

[29] Rosaasen N Taylor J Blackburn D Development and Validation of the Kidney Transplant Understanding Tool (K-TUT). Transplant Direct 2017;3: 10.1097/TXD.0000000000000647 PMC5367749 28361116

[30] Urstad KH Wahl AK Andersen MH Limited evidence for the effectiveness of educational interventions for renal transplant recipients. Results from a systematic review of controlled clinical trials. Patient Educ Couns 2013;90:147–54. 23199794 10.1016/j.pec.2012.10.020

[31] de Oliveira Marsicano E da Silva Fernandes N Colugnati F Transcultural adaptation and initial validation of Brazilian-Portuguese version of the Basel assessment of adherence to immunosuppressive medications scale (BAASIS) in kidney transplants. BMC Nephrol 2013;14:108. 23692889 10.1186/1471-2369-14-108 PMC3665586

[32] Chisholm MA Lance CE Williamson GM Development and validation of an immunosuppressant therapy adherence barrier instrument. Nephrol Dial Transplant 2004;20:181–8. 15572384 10.1093/ndt/gfh576

[33] Schulz KF Altman DG Moher D CONSORT 2010 statement: updated guidelines for reporting parallel group randomised trials. J Clin Epidemiol 2010;63:834–40. 20346629 10.1016/j.jclinepi.2010.02.005

[34] Chan AW Tetzlaff JM Altman DG SPIRIT 2013 statement: defining standard protocol items for clinical trials. Ann Intern Med 2013;158:200–7. 23295957 10.7326/0003-4819-158-3-201302050-00583 PMC5114123

[35] Garcia MFFM Bravin AM Garcia PD Behavioral measures to reduce non-adherence in renal transplant recipients: a prospective randomized controlled trial. Int Urol Nephrol 2015;47:1899–905. 26377496 10.1007/s11255-015-1104-z

[36] Montazeri A Vahdaninia M Mousavi SJ The 12-item medical outcomes study short form health survey version 2.0 (SF-12v2): a population-based validation study from Tehran, Iran. Health Qual Life Outcomes 2011;9:12. 21385359 10.1186/1477-7525-9-12 PMC3063185

[37] Bender BG Apter A Bogen DK Test of an interactive voice response intervention to improve adherence to controller medications in adults with asthma. J Am Board Fam Med 2010;23:159–65. 20207925 10.3122/jabfm.2010.02.090112

[38] Calder LA Cwinn AA Gatien M The feasibility of an interactive voice response system (IVRS) for monitoring patient safety after discharge from the ED. Emerg Med J 2017;35:180–5. 29175877 10.1136/emermed-2016-206192

[39] Castle T Cunningham MA Marsh GM Antidepressant medication adherence via interactive voice response telephone calls. Am J Manag Care 2012;18:e346–55. 23009333

[40] Heapy AA Higgins DM Goulet JL Interactive voice response-based self-management for chronic back pain: the COPES noninferiority randomized trial. JAMA Intern Med 2017;177:765–73. 28384682 10.1001/jamainternmed.2017.0223 PMC5818820

[41] Hettema JE Hosseinbor S Ingersoll KS Feasibility and reliability of interactive voice response assessment of HIV medication adherence: research and clinical implications. HIV Clin Trials 2012;13:271–7. 23134627 10.1310/hct1305-271 PMC3645257

[42] Kassavou A Sutton S Reasons for non-adherence to cardiometabolic medications, and acceptability of an interactive voice response intervention in patients with hypertension and type 2 diabetes in primary care: a qualitative study. BMJ Open 2017;7:e015597. 10.1136/bmjopen-2016-015597 PMC5724082 28801402

[43] Skolarus LE Piette JD Pfeiffer PN Interactive voice response-an innovative approach to post-stroke depression self-management support. Transl Stroke Res 2017;8:77–82. 27394917 10.1007/s12975-016-0481-7 PMC5507192

[44] Cizmic A Heilmann RM Milchak JL Impact of interactive voice response technology on primary adherence to bisphosphonate therapy: a randomized controlled trial. Osteoporos Int 2015;26:2131–6. 25956282 10.1007/s00198-015-3116-z

[45] Derose SF Green K Marrett E Automated outreach to increase primary adherence to cholesterol-lowering medications. JAMA Intern Med 2013;173:38–43. 23403978 10.1001/2013.jamainternmed.717

[46] Migneault JP Dedier JJ Wright JA A culturally adapted telecommunication system to improve physical activity, diet quality, and medication adherence among hypertensive African–Americans: a randomized controlled trial. Ann Behav Med 2012;43:62–73. 22246660 10.1007/s12160-011-9319-4 PMC8996679

[47] Vollmer WM Feldstein A Smith DH Use of health information technology to improve medication adherence. Am J Manag Care 2011;17:S79. PMC3641901 22216772

[48] Tsoli S Sutton S Kassavou A Interactive voice response interventions targeting behaviour change: a systematic literature review: Stergiani Tsoli. European Journal of Public Health 2018;28 suppl_4:cky213-167. 10.1136/bmjopen-2017-018974 PMC5855236 29478016

