# Supplementary material for: Effect of an interactive voice response system on self-management in kidney transplant recipients: Protocol for a randomized controlled trial
Source: Medicine (Baltimore). 2019 Feb 8;98(6):e14291. doi: 10.1097/MD.0000000000014291 (PMC6380874; doi:10.1097/MD.0000000000014291)
Supplement: Supplemental Digital Content [file medi-98-e14291-s001.doc]

Supplemental File 1 Characteristics of specialists in expert group (focus group and Delphi)

| **Expertise** | **Number** | **Gender** | **Age group (year)** | **Academic Certificate** |
| --- | --- | --- | --- | --- |
| Nephrologist | 4 | Male(n=0)  Female(n=4) | 40-50(n=3)  50-60(n=1) | Assistant Professor (n=3)  Professor (n=1) |
| Patient education nurses | 4 | Male(n=2)  Female(n=2) | 30-40 (n=1)  40-50(n=1)  50-60(n=2) | Bachelor degree(n=2)  Master degree(n=2) |
| Medical informatics | 2 | Male(n=1)  Female(n=1) | 30-40(n=1)  40-50(n=1) | Associate Professor (n=1)  PhD student (n=1) |

Supplemental File 2

Screenshots of IVRS (in Farsi)


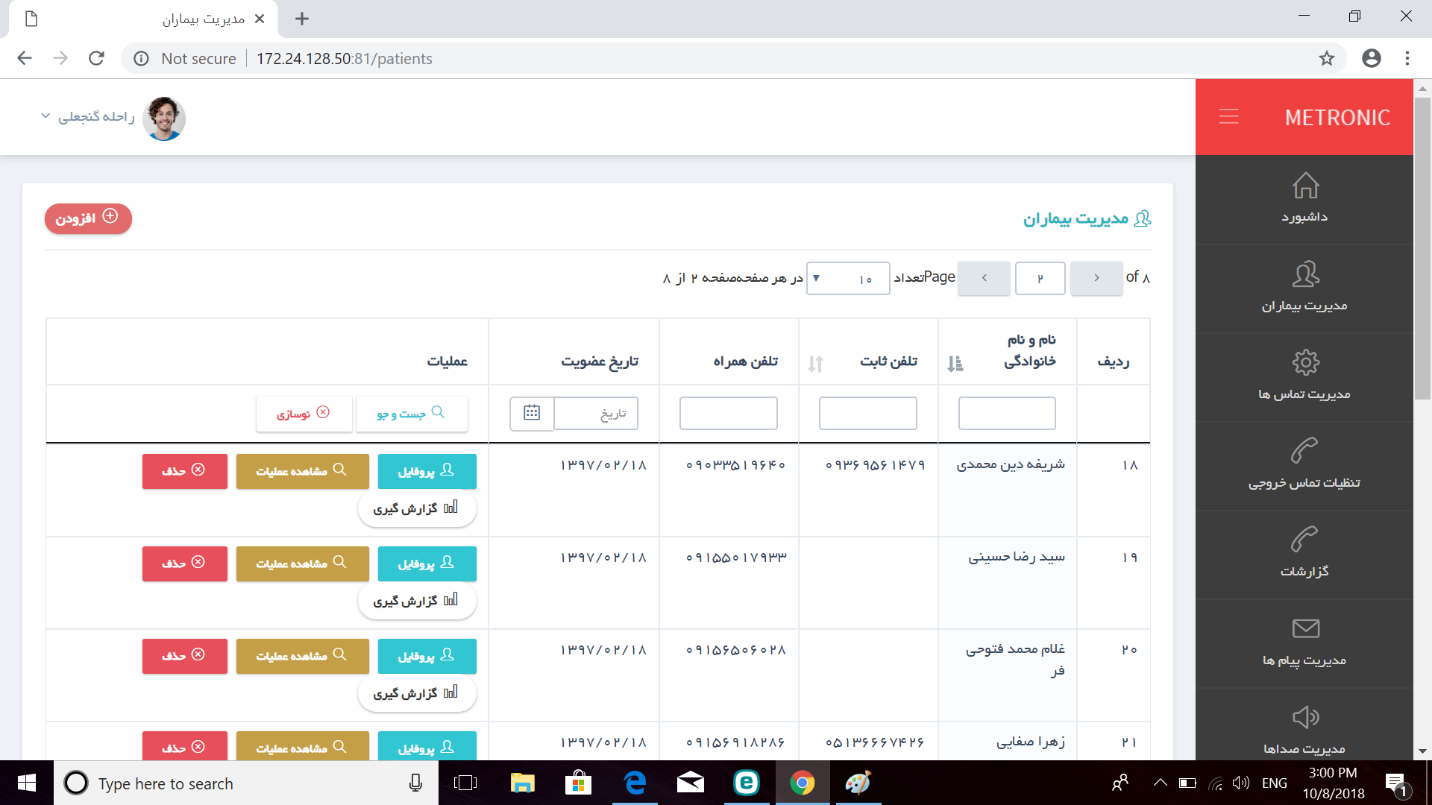


Figure 1. Patients management


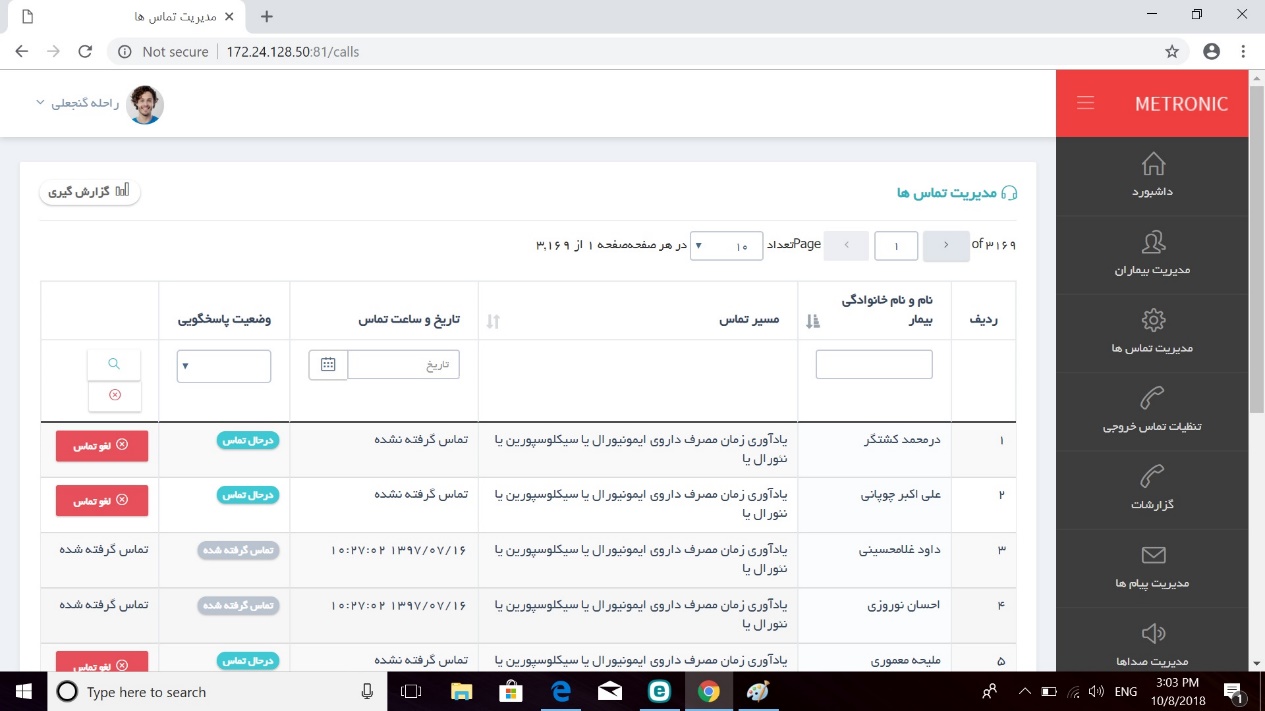


Figure 2. Call management


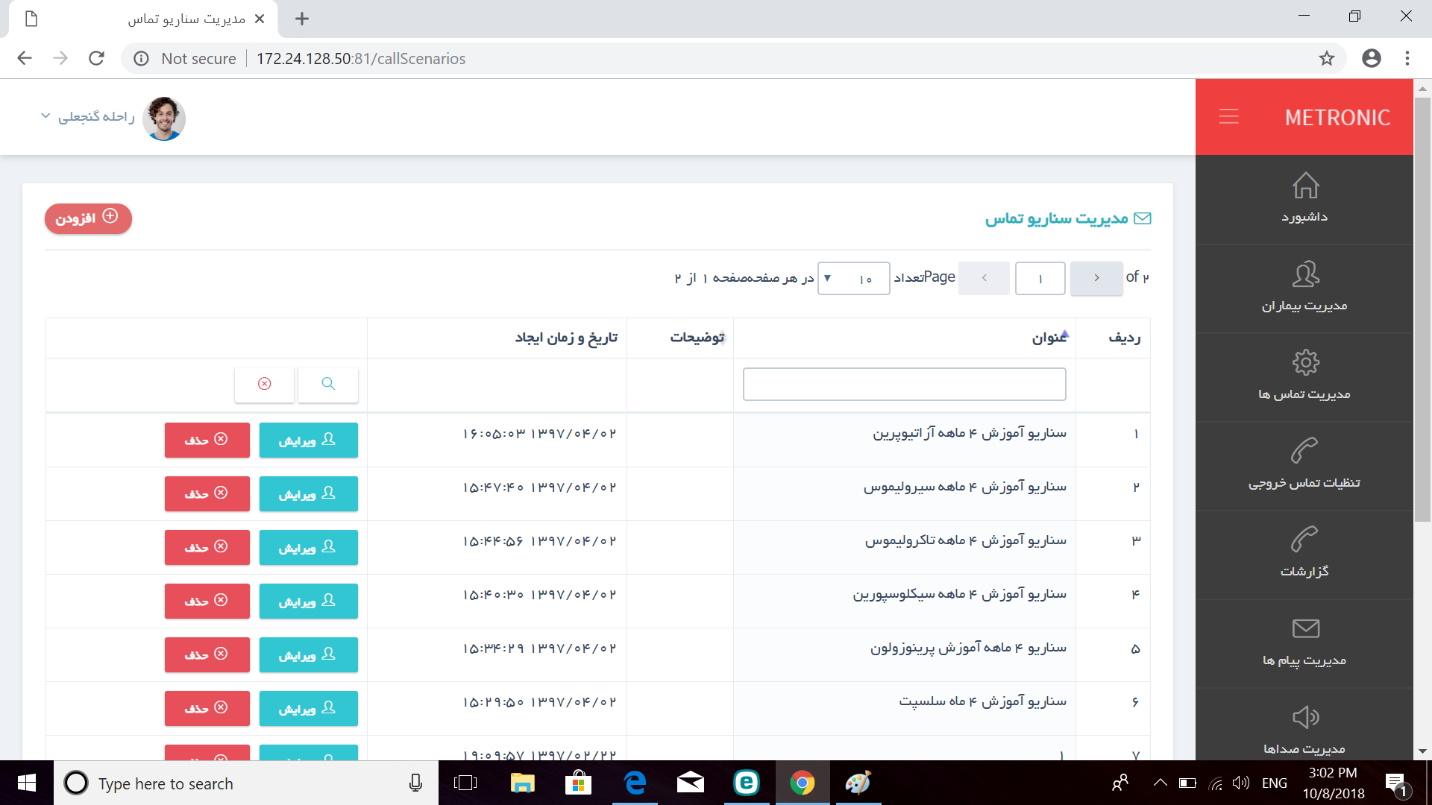


Figure 3. Scenario call management


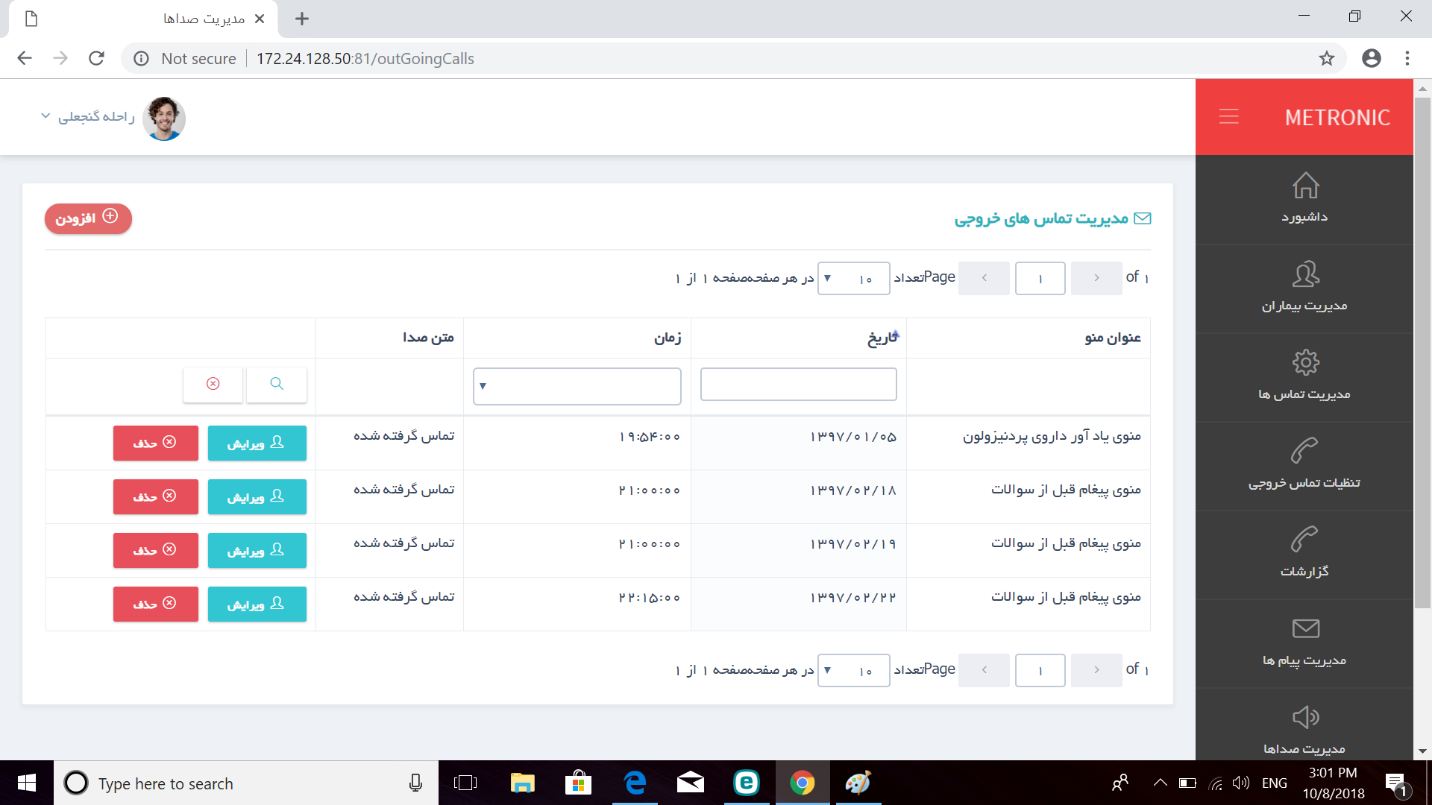


Figure 4. Outgoing calls management


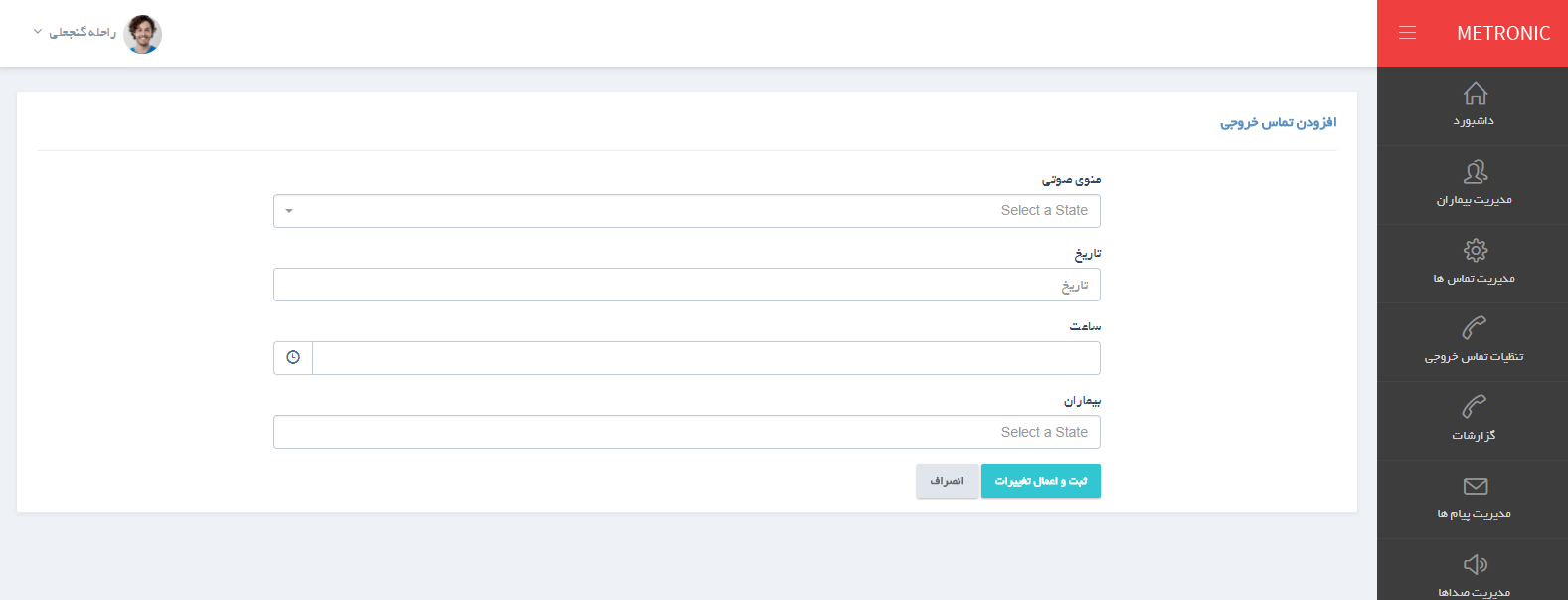


Figure 5. Call setting


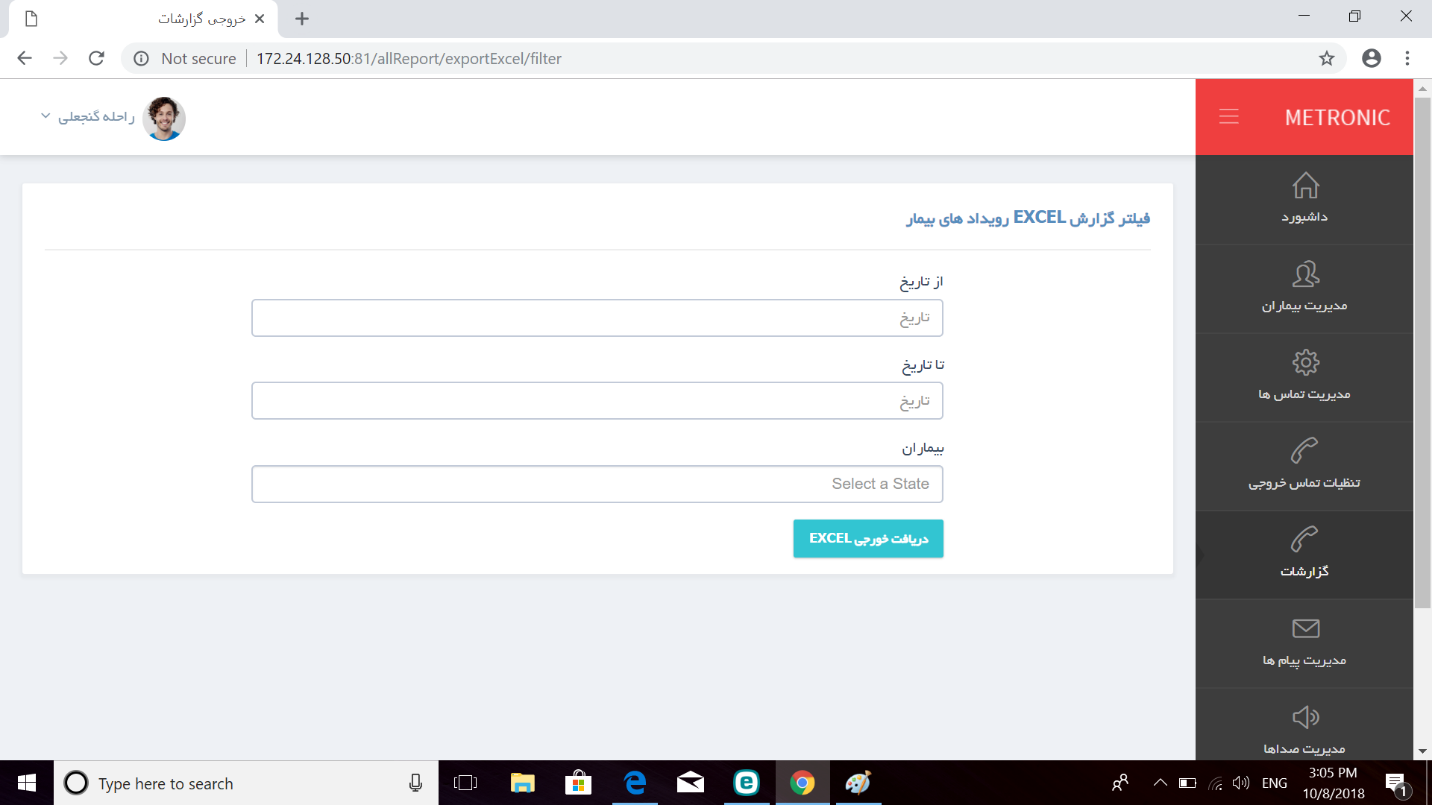


Figure 6. Patient calls reports


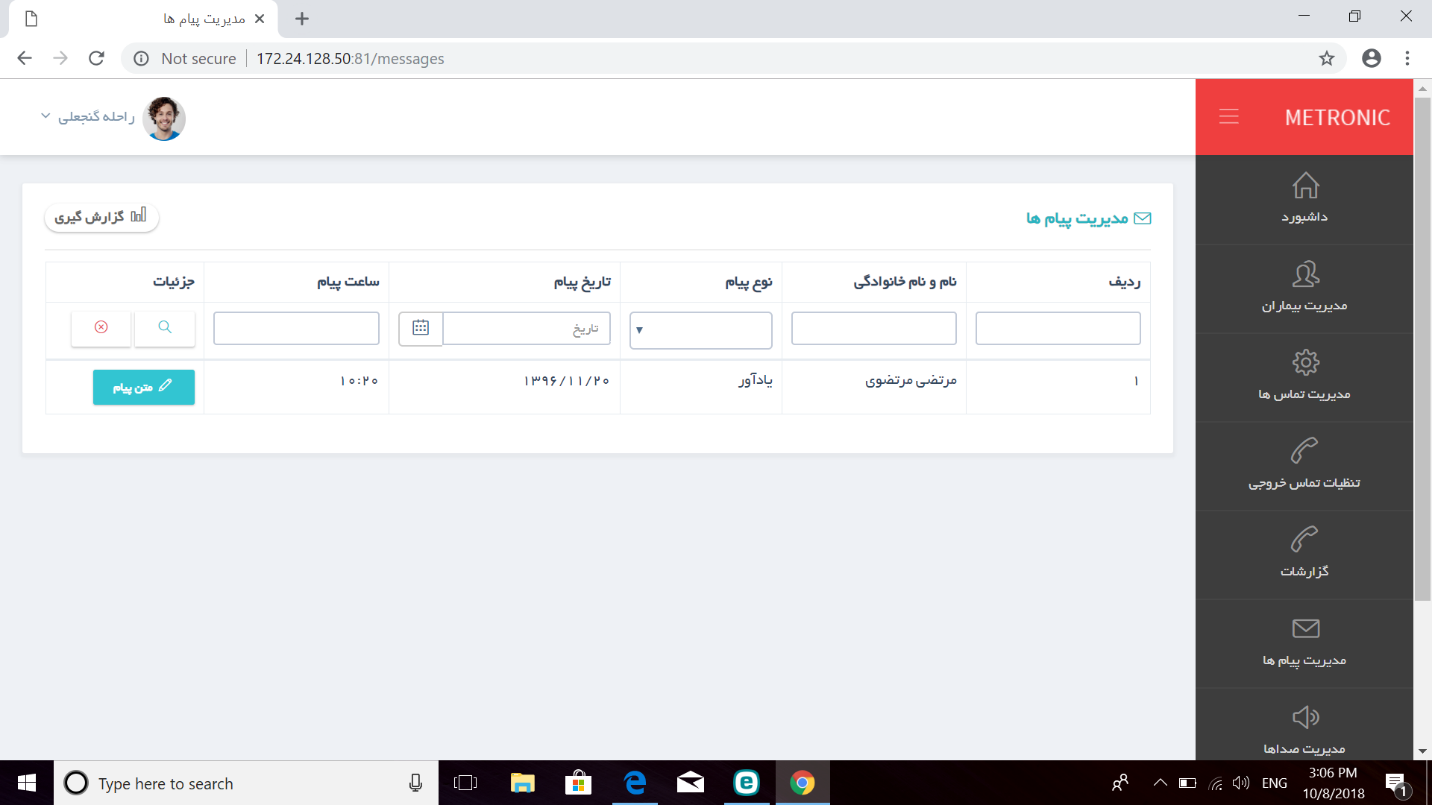


Figure 7. SMS management


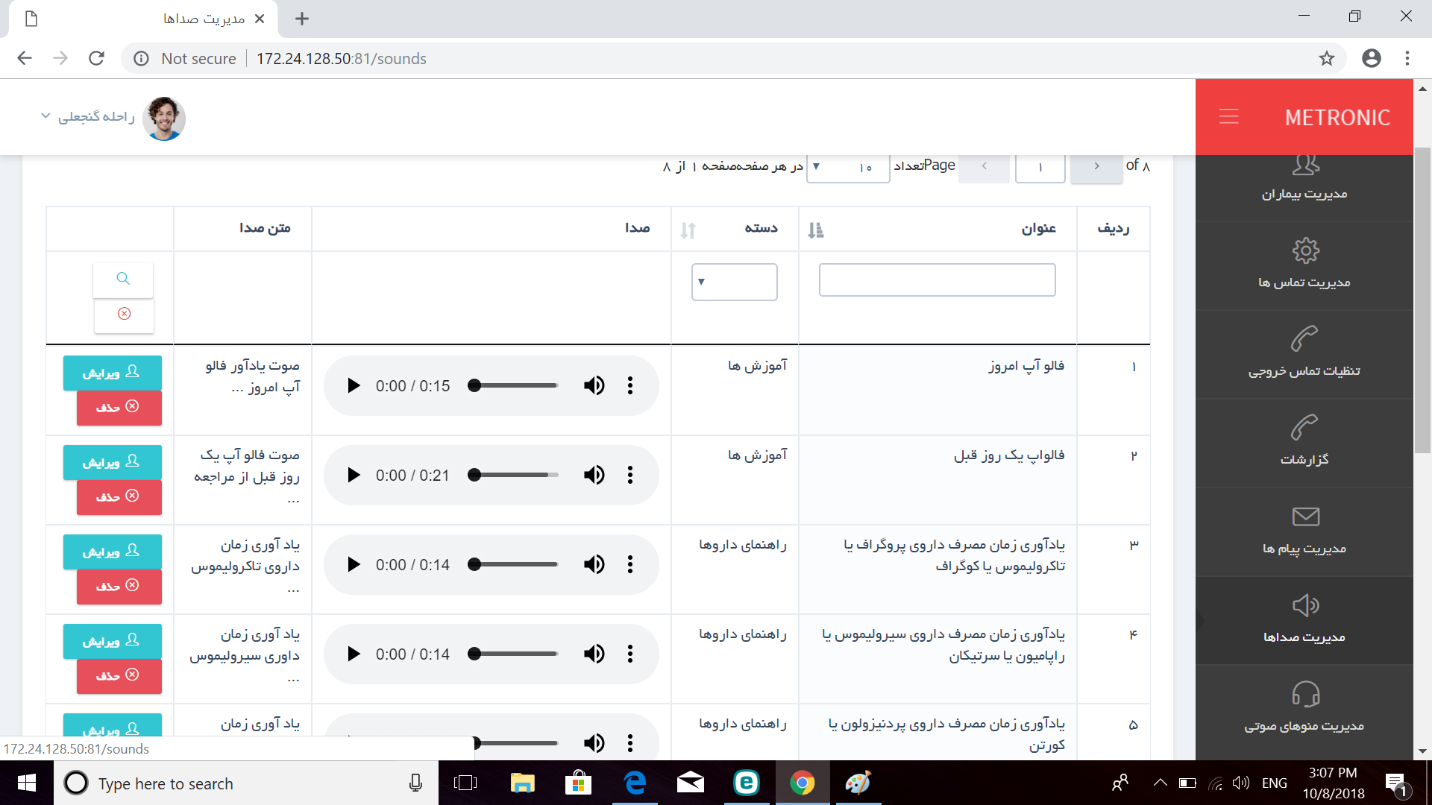


Figure 8. Voice management


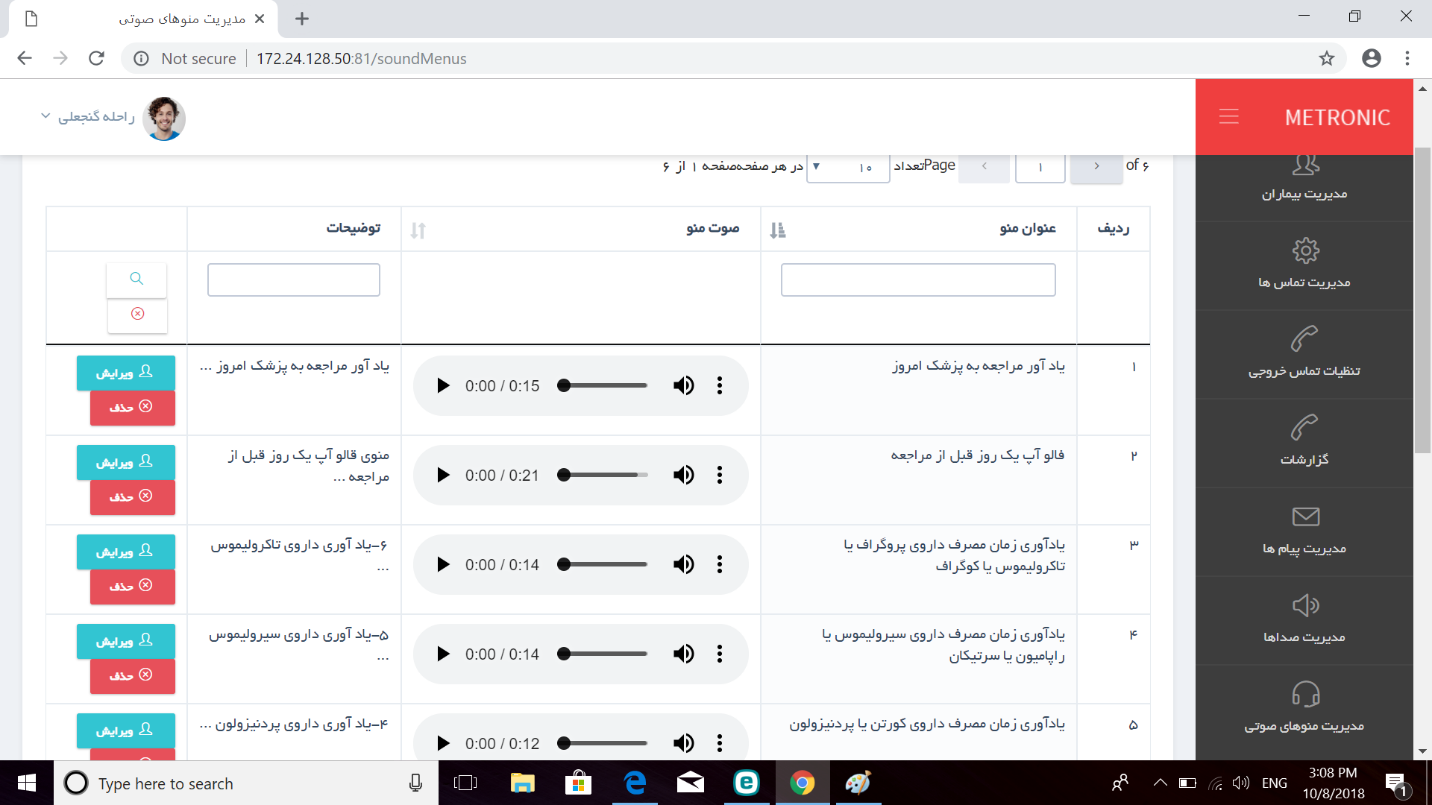


Figure 9. Voice menu management

Supplemental File 3 Complete list of questions presented to KT recipients.

| No | Category | Questions |
| --- | --- | --- |
| 1 | Satisfaction | How did you feel when you received a call from IVRS? |
| 2 |  | Do you prefer IVRS calls or in-person clinic education? |
| 3 |  | Are you satisfied with IVRS calls? |
| 4 | Problem | Was IVRS able to correct your behavior? |
| 5 |  | Do you have any problems completing IVRS calls? |
| 6 | Ease of use | Was it easy for you to use the IVRS? |
| 7 |  | Which type of IVRS calls did you find disturbing? |
